# Evaluating the safety and efficacy of the leukotriene receptor antagonist montelukast as adjuvant therapy in obese patients with type 2 diabetes mellitus: A double-blind, randomized, placebo-controlled trial

**DOI:** 10.3389/fphar.2023.1153653

**Published:** 2023-04-11

**Authors:** Eman El-Khateeb, Eman I. El-Berri, Esraa M. Mosalam, Mohamed Z. Nooh, Shimaa Abdelsattar, Amira M. Alghamdi, Sarah Alrubia, Mahmoud S. Abdallah

**Affiliations:** ^1^ Department of Clinical Pharmacy, Faculty of Pharmacy, Tanta University, Tanta, Al-Gharbia, Egypt; ^2^ Certara UK Limited (Simcyp Division), Sheffield, United Kingdom; ^3^ Department of Biochemistry, Faculty of Pharmacy, Menoufia University, Shebin ElKoum, Egypt; ^4^ Department of Internal Medicine, Faculty of Medicine, Menoufia University, Shebin ElKoum, Egypt; ^5^ Department of Clinical Biochemistry and Molecular Diagnostics, National Liver Institute, Menoufia University, Shebin ElKoum, Egypt; ^6^ Department of Biochemistry, Faculty of Science, King Abdulaziz University, Jeddah, Saudi Arabia; ^7^ Pharmaceutical Chemistry Department, College of Pharmacy, King Saud University, Riyadh, Saudi Arabia; ^8^ Department of Clinical Pharmacy, Faculty of Pharmacy, University of Sadat City, Sadat City, Menoufia, Egypt

**Keywords:** montelukast, T2DM, obesity, leukotriene receptor antagonist, metformin, diabetes

## Abstract

**Background:** Type 2 diabetes mellitus (T2DM) is common with obesity. Metformin is a first-line therapy for this condition. However, it has only a minor impact on weight loss in some patients.

**Aim:** This study aimed to evaluate the effectiveness, tolerability, and safety of combining montelukast therapy with metformin in obese diabetic patients.

**Methods:** One hundred obese diabetic adult patients were recruited and randomized into two equal groups. Group 1 received placebo plus metformin 2 g/d, and Group 2 received 2 g/d metformin plus 10 mg/d montelukast. Demographic, anthropometric measurements (e.g., body weight, body mass index [BMI], and visceral adiposity index), lipid profile, diabetes control measures (fasting blood glucose, glycated hemoglobin [HbA1c], and homeostatic model assessment for insulin resistance [HOMA-IR]), adiponectin, and inflammatory markers (e.g., TNF-α, IL-6, and leukotriene B4) were assessed and reported for each group at baseline and after 12 weeks of treatment.

**Results:** Both interventions significantly reduced all the measured parameters, except for adiponectin and HDL-C, levels of which increased compared to baseline data (*p* < 0.001). The montelukast group significantly improved in all parameters compared to the placebo group (ANCOVA test *p* < 0.001). The percentage changes in BMI, HbA1c, HOMA-IR, and inflammatory markers were 5%, 9%, 41%, and 5%–30%, respectively, in the placebo group compared to 8%, 16%, 58%, and 50%–70%, respectively, in the montelukast group.

**Conclusion:** Montelukast adjuvant therapy was superior to metformin-only therapy in diabetes control and weight loss, most likely due to its increased insulin sensitivity and anti-inflammatory properties. The combination was tolerable and safe throughout the study duration.

**Clinical Trial Registration**: [Clinicaltrial.gov], identifier [NCT04075110].

## 1 Introduction

Obesity increases susceptibility to insulin resistance (IR) and type 2 diabetes (T2DM). Nearly 70% of obese patients (with body mass index [BMI] ≥ 30) show IR, and more than 90% of T2DM patients are overweight, with BMI ≥25 (Whitmore, 2010; Calori et al., 2011). Obesity and metabolic comorbidities are thought to increase the risk of diabetes through several mechanisms (Blüher et al., 2020). One of these mechanisms is inflammation, specifically chronic low-grade inflammation (Pereira and Alvarez-Leite, 2014), which results from the accumulation of fat cells in the body, particularly in the abdominal area, and leads not only to increased release of pro-inflammatory cytokines but also to reduced anti-inflammatory and insulin-sensitizing adipokines such as adiponectin (Ferrante, 2007; Elks and Francis, 2010). Insulin resistance is another suggested key factor in the development of T2DM that can be a consequence of obesity-induced inflammation (Wondmkun, 2020), resulting in a lack of sensitivity toward secreted insulin and elevated blood sugar levels, which are hallmarks of T2DM.

Metformin is a glucose-lowering medication that also has anti-inflammatory and anti-oxidative properties (Drzewoski and Hanefeld, 2021). Hyperglycemia-related inflammation and oxidative stress are linked to IR, and these abnormalities affect beta cells, causing secretory dysfunction and increased apoptosis, ultimately leading to the development and progression of T2DM (Bhatti et al., 2022). Metformin has been approved and is reported in many studies to reduce weight in overweight and obese diabetic and non-diabetic patients. Many mechanisms for this effect have been proposed, of which the most important mechanisms relate to enhancing insulin sensitivity (Yerevanian and Soukas, 2019) and to metformin’s ability to control inflammation, lipid metabolism, oxidation, and deposition in the liver, skeletal muscles, and fat tissues (Malin and Kashyap, 2014; Bai and Chen, 2021).

In addition, adiponectin reduction and classical inflammatory cascades such as 5-lipoxygenase (5-LOX) can potentiate adipose tissue inflammation in obese individuals (Martinez-Clemente et al., 2011; Neels, 2013a; Back et al., 2014). The enzyme 5-lipoxygenase converts arachidonic acid, a type of fatty acid released from the cell membrane during times of inflammation, into leukotrienes (LTs) (Rådmark et al., 2015). Leukotrienes are signaling molecules that play a role in inflammation, as described in animal models, including obese rodents (Chakrabarti et al., 2011; Mothe-Satney et al., 2012). The released LTs flow, in turn, into the bloodstream and bind to specific receptors on the surface of cells. This can lead to increased inflammation and vasoconstriction, as well as non-vascular smooth muscle contraction (Barnes and Smith, 1999; Rådmark et al., 2015). Studies have shown that the 5-LOX pathway is upregulated in obese individuals and that inhibiting 5-LOX can improve insulin sensitivity and reduce inflammation (Neels, 2013b). This suggests targeting the 5-LOX pathway as a potential therapeutic approach for the treatment of “diabesity,” a term that describes the health problems arising from combined obesity and T2DM (Martinez-Clemente et al., 2011; Filgueiras et al., 2015).

Cysteinyl leukotrienes (Cyst-LTs) are among the potent bioactive lipids generated by the 5-LOX pathway, provoking inflammation. Their inflammatory effects occur *via* acting on Cyst-LT receptors that are not only present in the spleen, white blood cells, macrophages, and smooth muscles in the lungs, but also in the pancreas (Capra, 2004). Some evidence has shown that these pancreatic receptors are responsible for reducing insulin secretion and regulating glucose metabolism (Guo et al., 2018). Cysteinyl leukotrienes can contribute to a systemic inflammatory reaction by inducing the release of numerous pro-inflammatory cytokines and adipokines, such as nuclear factor (NF)-kB, tumor necrosis factor-α (TNF-α), interleukin 6 (IL-6), monocyte chemotactic protein 1, and macrophage inflammatory protein 1 (Back et al., 2011; Filgueiras et al., 2015). At the adipose tissue level, activation of the 5-LOX pathway provokes the abovementioned pro-inflammatory cytokines, increases circulating free fatty acid concentrations (Chakrabarti et al., 2011; Mothe-Satney et al., 2012), and causes hepatic steatosis in obese experimental animal models (Horrillo et al., 2010; Filgueiras et al., 2015).

Montelukast is a leukotriene receptor antagonist that mainly works by blocking the Cyst-LT1-receptor, helping to reduce inflammation in the airways and in other parts of the body (Riccioni et al., 2007; Ghorbanzadeh et al., 2022). Montelukast is approved for relieving symptoms associated with seasonal allergies and as a maintenance therapy for asthma, with a recommended dose of 10 mg for patients aged 15 years and older as approved by the U.S. Food and Drug Administration (FDA) (Food and Administration D, 2020). It is important to note that montelukast acts on LTs rather than by inhibiting the production of prostaglandins. It also inhibits the infiltration of neutrophils, reduces oxidative stress, and controls the production and release of several inflammatory mediators (Tugtepe et al., 2007; Ibrahim et al., 2014; Farzan et al., 2017; Chen et al., 2022). Due to its anti-inflammatory properties, it has shown promising results in the management of many conditions such as inflammatory bowel diseases (El-Alali et al., 2021), non-alcoholic steatohepatitis (Abdallah et al., 2021), some cardiovascular disorders (Hoxha et al., 2020; Hoxha et al., 2021), COVID-19 (Camera et al., 2022; Khan et al., 2022), and central nervous system inflammation (Yates et al., 2022).

Moreover, montelukast has shown positive neuroprotective effects (Lai et al., 2014), both experimentally and clinically, which can help in controlling diabetic neuropathy and diabetic retinopathy, two common complications of diabetes mellitus (Bapputty et al., 2019; Pham et al., 2021). Most of these effects are thought to be related to the anti-inflammatory mechanisms mentioned previously as inflammation is strongly related to diabetes pathophysiology, predisposition, and complications (Tsalamandris et al., 2019). However, little is known about the effect of montelukast in diabetes control or its safety in diabetic patients. This wide-ranging use of montelukast, as well as the lack of an effective therapeutic option for metabolic syndrome, has created a need to investigate the role of montelukast as a potential adjuvant therapy for diabesity.

Consequently, the primary hypothesis of the current study is that the concurrent administration of montelukast and metformin for 12 weeks can improve diabetes measures like glycated hemoglobin (HbA1c), as well as obesity indicators such as BMI and visceral adiposity index (VAI) in obese diabetic patients. The study’s secondary hypothesis is that the combination therapy is safe, tolerable, and able to significantly counter disease pathophysiology by improving the levels of some serum inflammatory markers, namely, adiponectin, TNF-α, IL-6, and leukotriene B4 (LTB4).

## 2 Materials and methods

### 2.1 Patients and methods

#### 2.1.1 Study design and ethical approval

This study was designed as a prospective, randomized, placebo-controlled, double-blind study. Patients were recruited from the Department of Internal Medicine at Menoufia University Hospital, Egypt, between July 2021 and July 2022. The study was approved by the hospital’s Research Ethical Committee with approval code INTM1. The study’s registration ID on clinicaltrial.gov is NCT04075110. The study protocol accords with the ethical standards of the Helsinki Declaration in 1975. All recruited patients, or their legal representatives (for illiterate patients), provided written informed consent prior to being enrolled in the study after giving verbal approval.

#### 2.1.2 Study population, randomization, and blinding

During the recruitment period, 150 patients were screened. One hundred newly diagnosed patients with T2DM were randomized equally into two groups with the help of an independent pharmacist using the closed-envelope methods. Envelopes were labeled with specific codes for each treatment intervention: Group 1 (*n* = 50; control or placebo) received metformin (Glucophage® 1,000 mg XR tablet, MERCK SANTE, France) at 2 g/day plus placebo, and Group 2 (montelukast group; *n* = 50) received a combination of metformin (Glucophage® 1,000 mg XR tablet, MERCK SANTE, France) at 2 g/day and montelukast (Sedokast® 10 mg tablet; SEDICO, Egypt) at 10 mg once daily. The treatment protocol for enrolled patients started with an initial metformin dose of 1 g/day, taken orally with a meal. After 7 days, the daily dose was up-titrated to 2,000 mg/day for 12 weeks. The diabetologist involved was planned to be unblinded only if a patient’s trial medicine had an impact on urgent emergency therapy. However, this was not the case for any of the patients; therefore, the study was kept double-blinded. The frequency of adult obese patients aged >18 years old with BMI ≥30 kg/m^2^, from both sexes, who are newly diagnosed with T2DM with HbA1c, is between 7% and 10%. Patients suffering from any other inflammatory disease (cardiovascular, asthma, bone, etc.) or severe hepatic or renal disease, or who were pregnant or lactating females, were excluded.

### 2.2 Measurements

The primary outcomes were changes in obesity-related anthropometric measures (body weight and BMI) and diabetes control measures (HbA1c). Secondary outcomes involved changes in the lipid profile, metabolic functions (VAI and HOMA-IR), and serum levels of adiponectin, TNF-α, IL-6, and LTB4.

#### 2.2.1 Demographic and anthropometric measurements

Demographic data such as age, sex, and medication history were collected from all participants at baseline. Physical examinations were also performed to collect patients’ weight, height, waist circumference (WC), body fat (kg), and body fat percentage (BFP), as well as their BMI, which was calculated using Eq. 1:

$$BMI=\frac{Weight\;\left(kg\right)}{{Height}^{2}\;\left(m\right)}.$$ (1)

An individualized diet based on initial weight, ideal weight, and dietary patterns was given to each participant at the time of assessment. A limited-energy balanced diet was given (1,000–1,200 kcal per day for female participants and 1,500–1,800 kcal per day for male participants), and participants were advised to engage in physical activity for at least 150 min per week during the study. Patients received frequent follow-up visits and phone calls to ensure that they carefully adhered to this predefined diet and activity plan.

#### 2.2.2 Biochemical measurements

From each participant, approximately 10 mL of venous blood was withdrawn through antecubital venipuncture between 8:30 and 10:30 a.m. and following an overnight (12-h) fast, before and after the 12 weeks of the intervention. Two milliliters of collected blood were collected in tubes containing ethylenediaminetetraacetic acid (EDTA) for HbA1c % assessment of the whole blood. The remaining 8 mL of blood were centrifuged at 4,500 g for 15 min to separate the serum, which was then placed in Eppendorf tubes. The separated serum was divided into two parts. The first part was used for the immediate determination of fasting lipid profile, including total cholesterol (TC), low-density lipoprotein cholesterol (LDL-C), high-density lipoprotein cholesterol (HDL-C), triglycerides (TGs), and fasting blood glucose (FBG). The remaining serum was frozen at −80°C until the analysis of fasting insulin (FI), adiponectin, TNF-α, IL-6, and LTB4. All fasting patients were allowed to eat a meal with at least 75 g of carbohydrates, and another blood sample was then collected after 2 h to measure 2-h post-prandial blood sugar (2-h PBG).

The glucose oxidase method was used for FBG and the 2-h PBG assessment (Spinreact, Spain, Ref No: TK41011). Antigen and antibody agglutination for HbA1c % was assayed (Spinreact, Spain, Ref No: TLIS50). The enzymatic colorimetric method was used for TC and TG level measurements (Spinreact, Spain, Ref No: TK41021 and MX41031, respectively). High-density lipoprotein cholesterol (HDL-C) concentration was measured by the enzymatic colorimetric method, which is based on the selective elimination of all lipoproteins other than the HDL-C fraction (Spinreact, Spain, Ref No: 1001095). The Friedewald formula (Eq. 2) was used for LDL-C calculation when TG levels were less than 400 mg/dL (Friedewald et al., 1972):

$$LD\mathrm{L-}C=TC-HD\mathrm{L-}C-\left(TG/5\right).$$ (2)

Serum levels of FI, adiponectin, TNF-α, IL-6, and LTB4 were assayed using commercial ELISA kits (MyBioSource, Inc., Cat No: MBS704195, MBS720699, MBS267654, MBS355306, and MBS3800318, respectively).

#### 2.2.3 Calculation of visceral adiposity index (VAI) and homeostatic model assessment for insulin resistance (HOMA-IR)

The VAI used for fat distribution estimation was sex-dependent and calculated by the formulas in Eqs 3, 4 (Amato et al., 2010).

For males,

$$VAI=(WC\;\left(cm\right)/\;\left(39.68+\left(1.88\times BMI\right)\right)\times \left(TG/1.03\right)\times \left(1.31/\;HDL-C\right).$$ (3)

For females,

$$VAI=(WC\;\left(cm\right)/\;\left(36.58+\left(1.89\times BMI\right)\right)\times \left(TG/0.81\right)\times \left(1.52/\;HDL-C\right).$$ (4)

The HOMA-IR index used for IR estimation is calculated as the fasting insulin level (μU/L) multiplied by fasting blood glucose (mmol/L), divided by 22.5 or by 405 when expressing fasting blood glucose in (mg/dL) mass units (Matthews et al., 1985).

### 2.3 Assessment of participants’ adherence, side effects, and drug tolerance

Montelukast and placebo tablets were given each month, and the participants’ adherence was assessed by their medication-refilling rate. Telephone calls every week and direct meetings every month were used for participant follow-up, the evaluation of their adherence, and the reporting of any adverse effects related to the drug, using a checklist. Non-adherence led to exclusion from the study when the participant used less than 90% of the study drugs or missed any monthly follow-up meetings.

### 2.4 Sample size calculation

The sample size was calculated with a predicted detectable mean difference ± standard deviation (SD) of 2 kg ± 3 for the total body weight (Ejtahed et al., 2019), or a mean difference ± SD of 0.5 ± 0.8 for HbA1c (Dagogo-Jack et al., 2018), between the two groups, with an alpha error of 0.05 and a beta error of 0.2. An attrition rate of 20% was estimated, providing 80% power with an effect size (Cohen’s d) ≥ 0.6 and a two-sided significance level of 5%. A sample size of 50 in each group was considered enough to detect any potential benefits of montelukast for weight loss or HbA1c reduction relative to placebo.

### 2.5 Statistical analysis

The statistical analysis was performed with IBM©SPSS® Statistics version 28.0 (SPSS Inc., September 2022, USA). The statistical analysis combined an alpha error of 0.05 with a 95% confidence interval and significance level of *p* < 0.05. The Shapiro–Wilk and Kolmogorov–Smirnov tests were used for testing the normal distribution of continuous variables. Quantitative variables were expressed as mean ± SD and qualitative variables were presented as numbers and ratios. Paired and two-sample *t*-tests were used for continuous quantitative data testing, and the chi-squared test for qualitative variables; the percentage (%) change was reported. The one-way analysis of covariance (ANCOVA) model was used for the primary and continuous secondary endpoint analysis, considering both treatments and sex as factors; the covariate was the baseline value of the endpoint. The relation between measured variables was determined using the Pearson correlation test. Figures were created using GraphPad Prism v9.

## 3 Results

### 3.1 Patients’ demographic data and clinical characteristics

**FIGURE 1:**
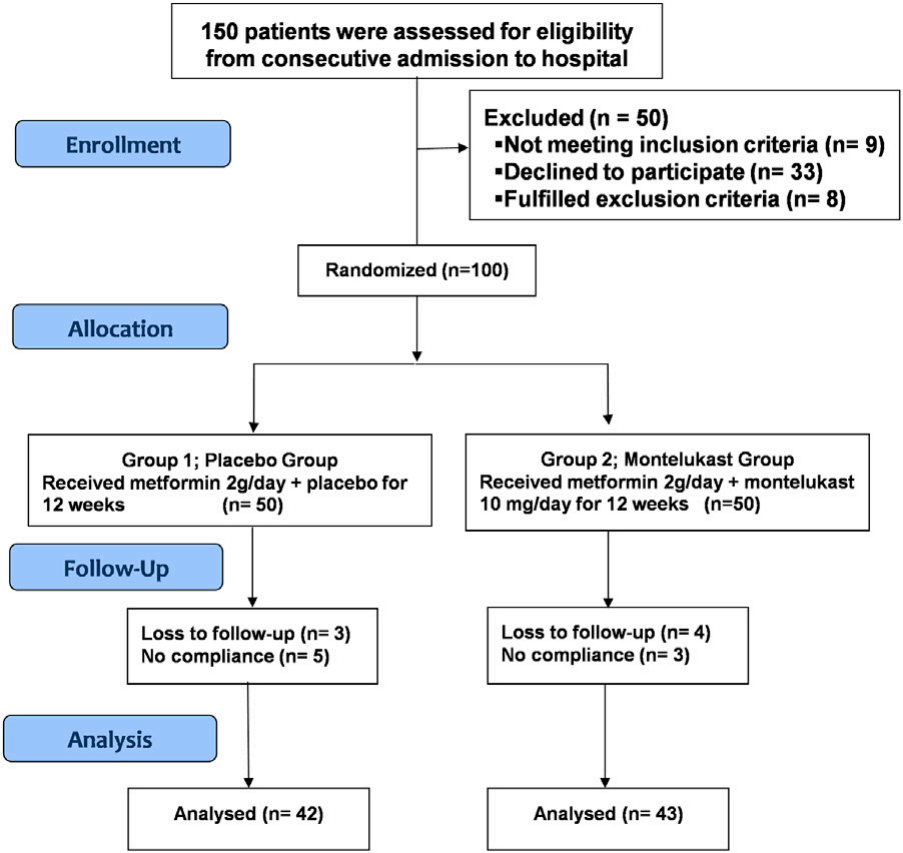
Patient flowchart.

Among patients who underwent randomization and follow-up, only 85 completed the 12 weeks of treatment and had their data analyzed (42 patients in the placebo group and 43 in the montelukast group), as illustrated in Figure 1.

The mean ages of patients were 44.24 ± 3.84 and 45.11 ± 4.12 years in the placebo and montelukast groups, respectively. In the placebo group, the mean body weight (BW) was 90.58 ± 4.32 kg, the mean body mass index (BMI) was 32.28 ± 0.91 kg/m^2^, and the mean waist circumference (WC) was 100.34 ± 6.12 cm. In the montelukast group, the mean BW was 90.32 ± 3.79 kg, the mean BMI was 32.45 ± 0.89 kg/m^2^, and the mean WC was 100.84 ± 6.29 cm. The two-sample *t*-test revealed that there were no statistical differences between the two groups at baseline (*p* > 0.05), as shown in Table 1.

**TABLE 1:** Baseline demographic, anthropometric, and laboratory data for the two study groups.

Parameter	Group 1 (placebo) n = 50	Group 2 montelukast) n = 50	Statistical *p*-values
Age (years)	44.24 ± 3.84	45.48 ± 4.21	0.127
Sex (male)	24 (48%)	25 (50%)	0.841
Smoking	24 (48%)	25 (50%)	0.841
Weight (kg)	90.58 ± 4.32	90.32 ± 3.79	0.057
Height (cm)	167.50 ± 4.13	166.84 ± 4.12	0.425
BMI (kg/m^2^)	32.28 ± 0.91	32.45 ± 0.89	0.348
WC (cm)	100.34 ± 6.12	100.84 ± 6.29	0.688
Body fat (kg)	34.87 ± 5.26	34.77 ± 5.06	0.927
Body fat percentage (BFP)	38.54 ± 5.86	38.60 ± 6.03	0.962
FBG (mmol/L)	8.02 ± 0.54	8.21 ± 0.57	0.966
FI (µU/mL)	17.91 ± 2.17	17.95 ± 2.15	0.925
HOMA-IR	6.61 ± 0.91	6.55 ± 0.89	0.784
2 h PBG (mg/dL)	251.44 ± 16.05	264.92 ± 14.40	0.142
HbA1c %	8.66 ± 0.56	8.56 ± 0.47	0.341
TC (mg/dL)	212.82 ± 11.52	213.98 ± 9.50	0.584
TG (mg/dL)	142.72 ± 9.90	141.12 ± 13.52	0.501
HDL-C (mg/dL)	45.80 ± 3.02	44.50 ± 4.10	0.074
LDL-C (mg/dL)	138.48 ± 12.62	141.26 ± 10.30	0.294
VAI	4.95 ± 0.92	5.03 ± 1.04	0.751

Data are presented as mean ± SD, number, and percent. BFP, body fat percentage; BMI, body mass index; FBG, fasting blood glucose; FI, fasting insulin; HOMA-IR, homeostasis model assessment–insulin resistance; WC, waist circumference; 2 h PBG, 2-h post-prandial blood sugar; HbA1c, glycated hemoglobin; TC, total cholesterol; TG, triglyceride; HDL-C, high-density lipoprotein cholesterol; LDL-C, low-density lipoprotein cholesterol; VAI, visceral adiposity index. *p* < 0.05 is considered significant.

### 3.2 Effect on anthropometric measures

As shown in Table 2, after 12 weeks of treatment, a paired *t*-test revealed that the placebo group, compared to its baseline data, produced statistically significant reductions in the values of BW, BMI, WC, body fat, and BFP (*p* < 0.0001) with percentage changes of −5.02%, −4.96%, −2.81%, −9.43%, and −4.59%, respectively. The montelukast group, compared to its baseline data, produced a statistically significant reduction in the values of BW, BMI, WC, body fat, and BFP (*p* < 0.0001) with higher percentage changes of −7.82% and −8.28%, −6.07%, −28.08%, and −22.20%, respectively.

**TABLE 2:** Anthropometric and laboratory data on the two study groups before and after treatment.

Laboratory parameter	Group 1 (placebo) *n* = 42			Group 2 (montelukast) *n* = 43		
	Before treatment	12 weeks after treatment	Statistical values	Before treatment	12 weeks after treatment	Statistical values
Weight (kg)	90.62 ± 4.55	86.5 ± 4.84*	(*p* < 0.0001; 95% CI: 2.03, 3.34)	90.95 ± 3.583	83.26 ± 3.30*	(*p* < 0.0001; 95% CI: 3.23, 5.11)
BMI (kg/m^2^)	32.33 ± 0.93	30.68 ± 0.66*	(*p* < 0.0001; 95% CI: 1.85, 3.07)	32.51 ± 0.91	29.76 ± 1.00*	(*p* < 0.0001; 95% CI: 3.36, 5.31)
WC (cm)	100.40 ± 6.11	97.52 ± 5.87*	(*p* < 0.0001; 95% CI: 2.40, 3.88)	101.05 ± 6.24	94.72 ± 5.43*	(*p* < 0.0001; 95% CI: 1.85, 3.05)
Body fat (kg)	35.09 ± 5.24	31.58 ± 4.82*	(*p* < 0.0001; 95% CI: 1.88, 3.12)	35.01 ± 5.18	25.01 ± 1.78*	(*p* < 0.0001; 95% CI: 1.55, 2.62)
Body fat percentage (BFP)	38.76 ± 5.86	36.77 ± 5.68*	(*p* < 0.0001; 95% CI: 1.85, 3.07)	38.60 ± 6.22	30.02 ± 1.65*	(*p* < 0.0001; 95% CI: 1.13, 2.03)
FBG (mmol/L)	8.13 ± 0.56	5.58 ± 0.41*	(*p* < 0.0001; 95% CI: 2.69, 4.32)	8.16 ± 0.57	5.19 ± 0.40*	(*p* < 0.0001; 95% CI: 3.14, 4.97)
FI (µU/mL)	17.91 ± 2.17	15.15 ± 2.28*	(*p* < 0.0001; 95% CI: 0.69, 1.45)	17.95 ± 2.15	11.76 ± 1.35*	(*p* < 0.0001; 95% CI: 1.883, 3.103)
HOMA-IR	6.48 ± 0.90	3.76 ± 0.67*	(*p* < 0.0001; 95% CI: 1.96, 3.23)	6.51 ± 0.89	2.72 ± 0.41*	(*p* < 0.0001; 95% CI: 2.93, 4.65)
2 h PBG (mg/dL)	249.55 ± 13.52	195.14 ± 21.09*	(*p* < 0.0001; 95% CI: 1.99, 3.27)	245.77 ± 13.06	161.30 ± 11.69*	(*p* < 0.0001; 95% CI: 4.97, 7.75)
HbA1c %	8.61 ± 0.58	7.83 ± 0.51*	(*p* < 0.0001; 95% CI: 2.26, 3.68)	8.57 ± 0.50	7.15 ± 0.36*	(*p* < 0.0001; 95% CI: 3.12, 4.94)
TC (mg/dL)	213.14 ± 11.81	192.21 ± 9.44*	(*p* < 0.0001; 95% CI: 2.48, 4.01)	213.70 ± 10.06	166.95 ± 12.10*	(*p* < 0.0001; 95% CI: 2.21, 3.59)
TG (mg/dL)	142.48 ± 10.43	128.98 ± 7.76*	(*p* < 0.0001; 95% CI: 1.46, 2.51)	139.81 ± 12.22	108.33 ± 8.30*	(*p* < 0.0001; 95% CI: −5.39, −3.42)
LDL-C (mg/dL)	138.62 ± 12.60	116.56 ± 11.33*	(*p* < 0.0001; 95% CI: 2.40, 3.88)	140.94 ± 10.71	87.43 ± 12.35*	(*p* < 0.0001; 95% CI: 2.48, 3.99)
HDL-C (mg/dL)	46.02 ± 2.96	49.86 ± 3.03*	(*p* < 0.0001; 95% CI: −1.48, −0.71)	44.79 ± 4.12	57.86 ± 2.67*	(*p* < 0.0001; 95% CI: −4.37, −2.74)
VAI	4.92 ± 0.94	4.11 ± 0.66*	(*p* < 0.0001; 95% CI:1.30, 2.28)	4.92 ± 1.01	2.89 ± 0.48*	(*p* < 0.0001; 95% CI: 2.28, 3.69)

Data are presented as mean ± SD, number, and percent. BFP, body fat percentage; BMI, body mass index; WC, waist circumference; 2-h PBG, 2-h post-prandial blood sugar; FBG, fasting blood glucose; FI, fasting insulin; HOMA-IR, homeostasis model assessment–insulin resistance; HbA1c, glycated hemoglobin; TC, total cholesterol; TG, triglyceride; HDL-C, high-density lipoprotein cholesterol; LDL-C, low-density lipoprotein cholesterol; VAI, visceral adiposity index. *p* < 0.05 is considered significant. * statistically significant compared to baseline. 95% CI**,** 95% confidence interval.

Furthermore, the montelukast group revealed a statistically significant reduction in BW and BMI after treatment, as compared to the placebo group, using the ANCOVA test [F (1, 80) = 67.003, *p*<0.0001, η2 = 0.456] and [F (1, 80) = 69.157, *p*<0.0001, η2 = 0.464], respectively. In addition, the montelukast group showed statistically significant reductions in the values of WC, body fat, and BFP after the intervention, as compared to the placebo group, using the ANCOVA test [F (1, 80) = 81.402, *p*<0.0001, η2 = 0.504], [F (1, 80) = 613.404, *p*<0.0001, η2 = 0.885], and [F (1, 80) = 1669.053, *p*<0.0001, η2 = 0.954], respectively.

The two-sample *t*-test revealed that there were no significant differences in the percentage changes for any anthropometric measures, between males and females, in either the placebo group or the montelukast group (*p* > 0.05). However, there were significant differences in the percentage change of WC (*p* = 0.023), body fat (*p* < 0.0001), and BFP (*p* < 0.0001) between males and females in the montelukast group.

### 3.3 Effect on diabetes control measures

Concerning blood glucose results, the placebo group produced statistically significant reductions, compared to its baseline data, in the levels of FBG, FI, PBG, HOMA-IR, and HbA1c % (*p* < 0.0001), with percentage changes of −30.90%, −15.42%, −22.39%, −41.14%, and −9.48%, respectively. Moreover, the montelukast group showed statistically significant reductions, compared to its baseline data, in the levels of FBG, FI, PBG, HOMA-IR, and HbA1c (*p* < 0.0001), with percentage changes of −37%, −34.48%, −34.67%, −58.53%, and −16.44%, respectively.

Furthermore, the montelukast group showed statistically significant reductions in the previously mentioned parameters, compared to the placebo group, after treatment, using the ANCOVA test [F (1, 80) = 18.839, *p* < 0.0001, η2 = 0.191], [F (1, 80) = 70.682, *p* < 0.0001, η2 = 0.469], [F (1, 80) = 72.698, *p* < 0.0001, η2 = 0.476], [F (1, 80) = 84.370, *p* < 0.0001, η2 = 0.513)], and [F (1, 80) = 143.184, *p* < 0.0001, η2 = 0.642], respectively. The intra- and inter-assay coefficients of variation for FI were 7.4% and 8.1%, respectively. In addition, the two-sample *t*-test revealed that there were no significant differences in the percentage changes in blood glucose measures between males and females, either in the placebo group or in the montelukast group (*p* > 0.05).

### 3.4 Effects on lipid profile

Regarding lipid profile, the placebo group, compared to its baseline data, produced statistically significant reductions in the levels of TC, TG, and LDL-C (*p* < 0.0001), with percentage changes of −9.68%, −9.63%, and −15.83%, respectively, and a significant rise in HDL-C level (*p* < 0.0001), with a percentage change of 8.86%. The montelukast group, compared to its baseline data, produced a statistically significant reduction in the levels of TC, TG, and LDL-C, with percentage changes of −21.98%, −23.24%, and −38.11%, respectively, and a significant rise in HDL-C level (*p* < 0.0001), with a percentage change of 30.02%.

Furthermore, the montelukast group showed statistically significant reductions in the lipid profile values of TC, TG, and LDL-C compared with the placebo group after treatment, using the ANCOVA test: [F (1, 80) = 129.530, *p* < 0.0001, η2 = 0.618], [F (1, 80) = 193.612, *p* < 0.0001, η2 = 0.708)], [F (1, 80) = 157.672, *p* < 0.0001, η2 = 0.663)], and [F (1, 80) = 211.456, *p* < 0.0001, η2 = 0.726)], respectively. In addition, the two-sample *t*-test revealed no significant differences in the percentage changes in lipid profile between males and females in either the placebo or the montelukast group (*p* > 0.05).

### 3.5 Effect on visceral adiposity index (VAI)

The results also revealed statistically significant reductions in VAI values compared to the baseline in both the placebo group (*p* < 0.0001) and the montelukast group (*p* < 0.0001), with percentage changes of −17.12% and −42.39%, respectively. In addition, there was a statistically significant decrease in the VAI value in the montelukast group compared with the placebo group after treatment, as determined by the ANCOVA test [F (1, 80) = 491.680, *p* < 0.0001, η2 = 0.860)]. Moreover, a two-sample *t*-test revealed no significant differences in the percentage changes of VAI between males and females in either the placebo or the montelukast group (*p* > 0.05).

### 3.6 Effect on adiponectin and inflammatory markers

As shown in Table 3, paired *t*-test results after 12 weeks of treatment showed that both the placebo and montelukast groups, compared to their baseline data, produced statistically significant increases in adiponectin levels and statistically significant decreases in TNF-α and IL-6 levels (*p* < 0.0001). The montelukast group, compared to their baseline data, produced a statistically significant decrease in LTB4 (*p* < 0.0001).

**TABLE 3:** Biochemical parameters of the two study groups before and after 12 weeks of treatment.

Laboratory parameter	Group 1 (placebo) *n* = 42			Group 2 (montelukast) *n* = 43			^b^Statistical values
	Before treatment	12 weeks after treatment	Statistical values	Before treatment	12 weeks after treatment	Statistical values	
Adiponectin (mcg/mL)	5.00 ± 0.79	7.20 ± 0.54^a^	(*p* < 0.0001; 95% CI: −2.85, −1.70)	5.12 ± 0.63	9.42 ± 0.86^a^	(*p* < 0.0001; 95% CI: −5.39, −3.42)	[F (1, 80) = 182.066, *p* < 0.0001, η2 = 0.695)]
TNF-α (pg/mL)	47.97 ± 8.08	34.08 ± 7.09^a^	(*p* < 0.0001; 95% CI: 2.31, 3.75)	47.01 ± 4.51	19.69 ± 3.13^a^	(*p* < 0.0001; 95% CI: 3.73, 5.86)	[F (1, 80) = 244.762, *p* < 0.0001, η2 = 0.737]
IL-6 (pg/mL)	46.19 ± 9.17	32.33 ± 7.68^a^	(*p* < 0.0001; 95% CI: 3.42, 5.42)	44.74 ± 10.05	20.13 ± 3.44^a^	(*p* < 0.0001; 95% CI: 2.12, 3.45)	[F (1, 80) = 182.455, *p* < 0.0001, η2 = 0.695)]
LTB4 (pg/mL)	23.47 ± 3.67	24.17 ± 2.27	(*p* = 0.51; 95% CI: −0.62, 0.002)	22.89 ± 4.02	8.05 ± 1.37^a^	(*p* < 0.0001; 95% CI: 3.25, 5.14)	[F (1, 80) = 2,851.275, *p* < 0.0001, η2 = 0.973)]

Data are presented as mean ± SD. TNF-α, tumor necrosis factor alpha; IL-6, interleukin-6; LTB4, leukotriene B4. *p* < 0.05 is considered significant; 95% CI**,** 95% confidence interval.

^a^ Statistically significant compared to baseline.

^b^ Statistically significant when two groups were compared after treatment.

In addition, the ANCOVA test showed that the montelukast group had statistically significant reductions in TNF-α, IL-6, and LTB4 levels compared with the placebo group after treatment, as well as a statistically significant increase in adiponectin levels (*p* < 0.0001).

Moreover, a two-sample *t*-test revealed no significant differences in the percentage changes of adiponectin and inflammatory markers between males and females, either in the placebo group or in the montelukast group (*p* > 0.05).

The intra- and inter-assay coefficients of variation for adiponectin were 8.3% and 9.6%; for TNF-α, 8% and 9.2%; for IL-6, 7.8% and 9.8%; and for LTB4, 8.4% and 9.4%, respectively.

### 3.7 Correlations among the investigated parameters

**FIGURE 2:**
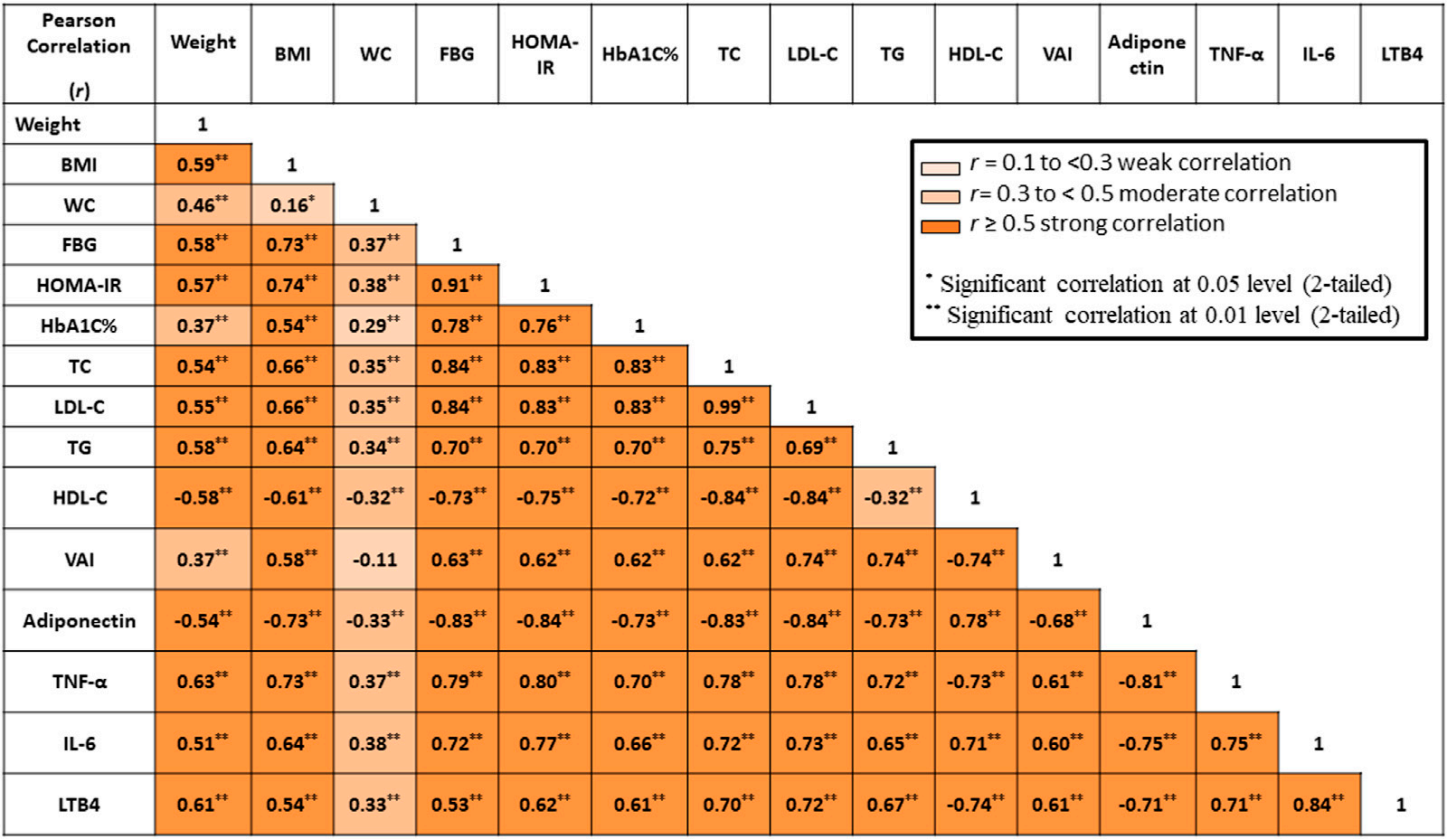
Correlation between all measured parameters. BMI, body mass index; WC, waist circumference; FBG, fasting blood glucose; FI, fasting insulin, HOMA-IR, homeostasis model assessment–insulin resistance; HbA1c, glycated hemoglobin; TC, total cholesterol; TG, triglyceride; HDL-C, high-density lipoprotein cholesterol; LDL-C, low-density lipoprotein cholesterol; VAI, visceral adiposity index; TNF-α, tumor necrosis factor alpha; IL-6, interleukin 6; LTB4, leukotriene B4. Color grades represent degrees of correlation.

As shown in Figure 2, the Pearson’s correlation test revealed statistically significant positive correlations between BMI and diabetes control measures **(**FBG, HOMA-IR, and HBA1c), as well as between BMI and VAI.

Concerning lipid profile, statistically significant positive correlations were reported between BMI and TC, LDL-C, and TG. In contrast, a statistically significant negative correlation was reported between BMI and HDL-C.

Regarding inflammatory markers, statistically significant positive correlations were observed between BMI and TNF-α, IL-6, and LTB4. In contrast, there was a statistically significant negative correlation between BMI and adiponectin.

Finally, at the end of the study, no statistically significant differences were found between the two groups concerning the reported side effects (Table 4).

**TABLE 4:** Reported side effects.

Side effect	Group 1(Placebo) (*n* = 42)	Group 2 (montelukast) (*n* = 43)	Statistical *p*-values
Abdominal distension	7 (16.67%)	8 (18.6%)	0.815
Abdominal pain (upper)	7 (16.67%)	8 (18.6%)	0.815
Constipation	6 (14.29%)	5 (11.63%)	0.715
Diarrhea	6 (14.29%)	5 (11.63%)	0.715
Decrease appetite	5 (11.90%)	6 (13.95%)	0.778
Nausea	3 (7.14%)	3 (6.98%)	0.976
Vomiting	3 (7.14%)	3 (6.98%)	0.976
Metallic taste in the mouth	5 (11.9%)	3 (6.98%)	0.437
Headache	3 (7.14%)	4 (9.3%)	0.717
Skin rash	0 (0%)	2 (4.76%)	0.157
Sore throat	0 (0%)	2 (4.76%)	0.157
Depressed mood	0 (0%)	2 (4.76%)	0.157

Data are presented as number and percentage. *p* < 0.05 is considered significant.

## 4 Discussion

Montelukast is a leukotriene receptor antagonist and therefore inhibits the inflammation cascade. It is mainly prescribed for inflammatory respiratory illnesses such as asthma. The current double-blinded, randomized, placebo-controlled clinical study is the first study showing both the usefulness of montelukast as adjuvant therapy in the management of T2DM (a non-respiratory disease) in obese patients and its potentiating effect on metformin for both body weight and the glycemic control level. It also provides proof of the hypothesized mechanism of LT-induced inflammation in developing obesity and insulin resistance.

Obesity is currently classified as a chronic inflammatory condition, with high levels of circulating cytokines in obese patients. This inflammatory reaction is thought to drive the progression of other comorbid conditions that occur with high severity in obese patients. T2DM is one of these prominent comorbidities, and the majority of such patients are obese (Grant et al., 2021). However, this study, along with previous studies, has proved that obesity is a manageable risk factor of diabetes and that weight loss medications are highly recommended for diabetic patients to reduce fat deposition on insulin receptors and to prevent or treat the condition of insulin resistance that is highly prevalent in diabetic patients.

The American Diabetes Association (ADA) recommends that metformin be used to treat people newly diagnosed with T2DM and that HbA1c levels be kept below or around 7%. If the HbA1c target is not achieved or maintained after 3–6 months of metformin monotherapy at the maximum tolerated dose (2000 mg), treatment modification with the addition of a second oral antihyperglycemic agent or the initiation of insulin is recommended (Association, 2020). The gradual addition of medications to metformin to keep A1C at target allows for a more accurate assessment of the positive and negative effects of new drugs and reduces patient risk and expenses (Association, 2020). However, there is evidence to support early combination therapy for the faster achievement of glycemic goals and later combination therapy for longer glycemic effect durability, particularly in patients with A1C levels 1.5%–2.0% above target (Phung et al., 2014). As a result, the dose was chosen to compare the maximum effect of metformin with and without montelukast. However, metformin monotherapy is not recommended by the Endocrine Society Practice Guideline on Pharmacology for Obesity for obese patients who do not have metabolic complications such as diabetes due to its modest and inconsistent effects (Apovian et al., 2015). This modest effect of metformin-only therapy produces a BMI reduction of nearly one unit on average, according to a meta-analysis in populations with different age groups, comorbidities, and treatment durations (Pu et al., 2020). The resulting reduction was also shown to recede at high grades of obesity and reached its peak effect within 6 months of treatment (Pu et al., 2020). The current study’s results are consistent with these findings as the reduction in mean BMI was around 1.65 units for diabetic patients in the placebo treatment group. However, the total reduction in average BW was only 4 kg, compared to the 8 kg reported in a similar study, which can be attributed to the shorter therapy duration herein (12 weeks) compared to the previous study’s duration of 24 weeks (Lee and Morley, 1998).

Therefore, in order to potentiate metformin’s weight loss effect, various anti-inflammatory drugs with different mechanisms were thought to have synergistic effects. Many drugs have been explored, such as Ginkgo biloba extract (Hussain et al., 2022), statins (Van Stee et al., 2018), and fibrates (Li et al., 2011). However, their effects on body weight are not clear, and some of these drugs (such as statins) have been associated with adverse effects when combined with metformin (Van Stee et al., 2018). Other drugs are being proposed as adjuvant therapies for metformin in obese diabetics, such as glucagon-like peptide 1 (GLP-1) receptor agonists (e.g., liraglutide and semaglutide), which increase insulin secretion from pancreatic cells and are therefore effective as antidiabetics (Lee, 2021). Moreover, they reduce satiety by acting on appetite centers and have been shown to have some effect on body fats (Zander et al., 2002). Similarly, cannabinoid receptor type 1 antagonists, such as rimonabant, have shown some anorexic effects, either alone or in combination with GLP-1 agonists in experimental animals (Xie et al., 2007). However, these agents have poor adherence due to the need for intravenous administration, their high cost, and the serious central nervous system side effects associated with their long-term use (Nguyen et al., 2019; Lee, 2021). Therefore, the current study aimed to offer a safer, pharmacoeconomic, and tolerable alternative.

In the current study, montelukast triggered an additional significant reduction in mean ratios to baseline for obesity indices such as BW, BMI, WC, and body fat. It has also shown improvements in the lipid profile. The adjuvant drug was able to push the reduction in average BMI to 2.75 units and the average total BW to 7.6 kg (approximately 1.5 times and 2 times higher than the metformin-only effect, respectively).

The reduction in BW by the concomitant administration of montelukast and metformin increased the anti-diabetic effect of metformin, as revealed by the greater reduction in HbA1c, 2-h PBG, and FBG levels in the montelukast group in comparison with the placebo, as well as the moderate-to-strong positive correlation between BMI and FBG, HbA1c, HOMA-IR, and VAI. This finding supports the hypothesis that weight loss enhances insulin sensitivity, as previously reported (Ikeda et al., 1996; Schenk et al., 2009; Clamp et al., 2017), which is also supported by the lower HOMA-IR and VAI levels in the combined therapy group.

In addition to the above-mentioned effects of weight reduction on diabetes control are the changes in the levels of inflammatory markers. High levels of inflammatory cytokines and adipokines associated with obesity are well known to contribute to insulin resistance (Clamp et al., 2017) and consequently to the progression of T2DM and metabolic syndrome in obese patients (King, 2008). Therefore, it was not surprising to notice better diabetes control after the significant declines in TNFα, IL-6, and LTB4 levels in the montelukast group compared to the placebo. Although metformin has been reported to have some anti-inflammatory effect consistent with this drop in inflammatory markers in the placebo group, montelukast most likely triggers this effect through Cyst-LT1-receptor antagonism.

Many studies have highlighted the systemic value of montelukast as an anti-inflammatory drug. Most of these studies attribute this effect to its ability to inhibit NF-kB activation in various cells, which has been shown to result in the lowered release of IL-6, MCP-1, and TNFα from blood monocytes of both control and asthmatic subjects induced by lipopolysaccharides (Maeba et al., 2005). It has also reduced the expression of IL-8 induced by TNFα in macrophage cell lines (Tahan et al., 2008) and IL-10 production in guinea pigs (Wu et al., 2006).

One of the most important proinflammatory cytokines involved in insulin resistance and obesity is LTB4 (Callegari and Oliveira, 2022). Unlike the allergy-inducing CysLTs, LTB4 potentiates the inflammatory cascade by acting on its specific receptors, leading to vasodilatation and the infiltration of neutrophils and other blood cells into various tissues, ultimately causing tissue injuries (Alomair et al., 2022; Marques et al., 2022). The current study’s findings show that montelukast does not only block LTB4 receptors but reduces the serum levels of this pro-inflammatory mediator compared to placebo. A possible mechanism of this effect is montelukast’s ability to inhibit LTB4 formation through the 5-LOX-arachidonic acid pathway, which adds to its anti-inflammatory effect, along with Cyst-LT1-receptor antagonism (Ramires et al., 2004; Wang et al., 2021). This finding agrees with the preclinical study by Tu et al., which reported a significant decline in serum LTB4 levels after oral administration of montelukast in rats (Tu et al., 2017).

Another key finding is the significant increase in adiponectin levels for the montelukast group compared to placebo and baseline levels. Adiponectin is known as the protective adipokine that is synthesized in adipose tissue. Its levels are known to negatively correlate with the severity of obesity and metabolic syndrome (Esfahani et al., 2015). Increased levels of adiponectin are also associated with increased insulin sensitivity and general anti-inflammatory reactions for many inflammatory conditions in addition to obesity, such as asthma (Coffey et al., 2015) and inflammatory bowel disease (Yamamoto et al., 2005).

The present study’s findings prove the indirect role of LTs in obesity and consequently in developing T2DM. Many studies have explored the potential role of non-steroidal anti-inflammatory drugs (NSAIDs) such as salicylic acid and its prodrug, salicylate (Faghihimani et al., 2013; Goldfine et al., 2013), as well as naproxen, cromolyn (Motawi et al., 2013), and celecoxib (Tian et al., 2014), in the management of diabetes and obesity. Despite the modest positive effects of these NSAIDs in controlling diabetes and obesity, as shown in these studies, they work by inhibiting prostaglandins and are therefore associated with many adverse events, such as gastric ulcers and renal impairment, which are critical in diabetic patients and impair patient compliance and long-term use. However, acting on the LTs/5-LOX pathway using therapeutic doses of montelukast has been shown to bypass these obstacles and improve its therapeutic benefits with no such adverse effects, as proven herein.

In addition to all the aforementioned positive effects of montelukast, the combination of metformin and montelukast was found to be tolerable, safe, and comparable to metformin-only therapy. Safety and tolerability were evaluated through the signs and symptoms of gastrointestinal discomfort, skin reactions, and mood disorders. This was consistent with earlier studies highlighting the safety and tolerability of montelukast in various populations (Benninger and Waters, 2009).

Although our study found montelukast to be safe and effective as an adjuvant in obese diabetic patients, it did have some limitations. All participants were classified as having class 1 obesity, or a BMI of 30–35, and higher levels of obesity were not tested. Moreover, the average HbA1c level after the combined therapy treatment was 7.15 ± 0.36, which did not meet the target of less than 7%. In addition, no data were collected on lifestyle and dietary patterns before the initiation of the study, making it difficult to determine how much these factors may have contributed to the findings. Given the short duration of therapy, long-term effects and side effects were not reported, as with other clinical studies of a similar kind, but more side effects may appear when the study is generalized to a larger population; these can be considered limitations to the current study. Furthermore, because this was the first clinical trial of montelukast in obese diabetic patients, we had no guide in making an unambiguous conclusion about the best dosage. In addition, its efficacy and safety in the presence of other comorbid conditions, such as renal and hepatic diseases, were not investigated. Larger clinical studies are recommended, especially in morbidly obese diabetic patients, with various dose regimens, and for longer durations of montelukast–metformin combination therapy.

## 5 Conclusion

The current randomized clinical study reveals the superiority of the montelukast–metformin combination over metformin-only treatment in obese patients with T2DM. The combined therapy was capable of significantly improving obesity, diabetes, inflammatory markers, and insulin resistance measures. Montelukast is a cheap, safe, tolerable, and effective adjunctive therapy for obese patients suffering from T2DM. Larger and longer clinical studies are recommended to trace the combination’s safety and efficacy in the long term.

## Data Availability

The raw data supporting the conclusion of this article will be made available by the authors, without undue reservation.
